# Supplementary material for: An integrated approach to identify bimodal genes associated with prognosis in câncer
Source: Genet Mol Biol. 2021 Oct 4;44(3):e20210109. doi: 10.1590/1678-4685-GMB-2021-0109 (PMC8495773; doi:10.1590/1678-4685-GMB-2021-0109)
Supplement: Table S1 - [file 1415-4757-GMB-44-3-e20210109-s1.pdf]

Genetics and Molecular Biology
https://doi.org/10.1590/1678-4685-GMB-2021-0109

"Supplementary Material to "An integrated approach to identify bimodal genes associated with prognosis in cancer"

Table S1 - List of bimodal genes estimated in each tumor.

Tumor	Bimodal genes	Total
**BLCA**	C5orf25/ C7orf4/ CYorf15A/ DDX3Y/ EIF1AY/ LRRC14/ MAGEA6/ MTAP	8
**BRCA**	DNALI1/ FKBP1AP1/ FOXA1/ GATA3/ MLPH/ PRR15/ RPS27/ RPS28/ SCNN1A/ SIDT1/ SLC35E2/ SLC44A4/ SPDEF	13
**CESC**	ANXA8L2/ C2orf43/ CCDC38/ GPR87/ H2AFY2/ PPP3R2/ RPS27/ SEPT2/ SLC35E2/ SMC1B/ TMEM40/ UPRT/ ZNF304/ ZNF586	14
**COAD**	C10orf113/ C19orf46/ C21orf54/ C5orf47/ CHFR/ CHMP7/ CYorf15A/ EIF1AY/ FAM127B/ RNLS/ RPS26P11/ SLC22A10/ SLC38A5/ SPAG16/ TAF15/ TP53/ TPI1P3/ UTY/ ZFY/ ZNF595/ ZNF681/ ZNF737	22
**ESCA**	ABCA2/ CHFR/ DDAH1/ ICA1/ SCNN1A/ SPZ1/ ZNF502/ ZNHIT2	8
**GMB**	ATXN10/ BAG1/ CYorf15A/ DDX3Y/ EIF1AY/ FAM138B/ FDX1L/ GPR52/ GPX8/ JMJD5/ KDM5D/ KLHL9/ L3MBTL2/ LOC90586/ MCAT/ NDN/ NLGN4Y/ RNF185/ RPS4Y1/ SLC35E2/ SMU1/ TMED10/ TMSB4Y/ TOX4/ TTTY15/ USP9Y/ UTY/ ZBTB45/ ZFY	29
**HNSC**	CDH3/ HOXB7/ SCRT2/ SLC35E2/ ZNF71	5
**KIRC**	CYorf15A/ EIF1AY/ FLJ40504/ GSTM1/ IFNA8/ LOC440173/ LQK1/ OR2A12/ PPBPL2/ RPL9/ RPS4Y1/ SLC35E2/ UTY/ XRRA1/ ZDHHC15	15
**KIRP**	AMT/ ARHGEF16/ C19orf46/ DYNC2LI1/ ERAP2/ FAM50B/ FUK/ psiTPTE22/ RPS27/ SLC35E2/ SNRPN	11
**LAML**	ADSL/ AUH/ GTSF1/ HES7/ HOXB6/ LOC220594/ LOC644172/ LOC654433/ MEIS1/ PAX8/ PCCA/ PRKY/ PSMD8/ SLC35E2/ SNRPN/ TTC26/ WNT9B/ ZFY	18

Tumor	Bimodal genes	Total
**LGG**	AK2/ ASAP3/ AWAT1/ BSDC1/ C19orf2/ C1orf109/ C1orf144/ CALCRL/ CARD8/ CCDC97/ CDC42/ CSDE1/ DDX20/ DDX3Y/ DNAJC8/ DRG2/ EIF1AY/ ERBB4/ FAM54B/ FBXO42/ FNBP1L/ FOXJ3/ GALNT13/ GNAI3/ GNL2/ GPBP1L1/ GPN2/ GRPEL2/ HDAC1/ HP1BP3/ IRX2/ KDM5D/ KIAA0319L/ KPNA6/ LOC253039/ LOC644145/ LRRC41/ LRRC42/ LSM14A/ MFN2/ MID2/ MIER1/ MLH3/ MT1IP/ MTHFR/ NADK/ NFIA/ NLGN4Y/ NRD1/ OSBPL9/ PARS2/ PHACTR4/ PHC2/ PPIE/ PRPF38A/ PRPF38B/ PSMB2/ RBBP4/ RPF1/ RPL9/ RPS27/ RPS28/ RPS4Y1/ S100PBP/ SCP2/ SERBP1/ SF3A3/ SFRS4/ SH3GLB1/ SLC35E2/ SNIP1/SNRNP40/ THRAP3/ TMEM167B/ TMEM69/ TTC4/ TTTY15/ TXNDC12/ USP48/ USP9Y/ UTY/ VRK3/ WASF2/ WDR77/ YTHDF2/ ZFY/ ZNF233/ ZNF576/ ZNF691/ ZNHIT6	90
**LIHC**	ALG8/ C3orf74/ CCDC79/ CYorf15A/ DDX3Y/ EIF1AY/ KDM5D/ OBSL1/ SLC35E2/ UTY/ ZFY	11
**LUAD**	CYorf15A/ GSPT2/ GSTM1/ OR51I1/ RPS27/ SLC35E2/ TMLHE	7
**LUSC**	AWAT1/ DNAJB8/ MTAP/ RPS27/ SLC35E2/ ZNF502	6
**OV**	GSTT1/ RCC2/ TP53/ ZNF37A/ ZNF597	5
**PAAD**	C9orf53/ CD164/ CIB3/ CYorf15A/ DMRTA1/ ERAP2/ FKBP1AP1/ GGT5/ GSTM1/ LOC285401/ RPS27/ SLC35E2	12
**PCPG**	ARFIP2/ ASFMR1/ ASH1L/ C11orf58/ C14orf21/ CACNA1B/ CCBL2/ CDKL4/ CECR1/ CKAP5/ COPS3/ CPEB1/ CSNK2A1P/ CYorf15A/ DDX3Y/ DLAT/ EIF1AY/ EIF2B5/ EXOSC10/ FBXO3/ FLII/ GGNBP2/ GMCL1/ GMIP/ HTR3B/ KDM5D/ LMTK2/ MAN1C1/ METT5D1/ METTL6/ MLH1/ NHS/ OSBPL6/ PDCD10/ PEX19/ PRKY/ RGP1/ RPL36A/ RPS4Y1/ SENP3/ SHPK/ SHPRH/ SLC35E2/ TBC1D14/ TGFBRAP1/ TMEM38B/ TOLLIP/ TRIM3/ TUBGCP4/ USP9Y/ ZC3H13/ ZFY/ ZNF665	53
**PRAD**	CHMP7/ ERAP2/ LQK1/ OR2AG2/ RPL9/ RPS27/ RPS28/ SLC35E2/ XRRA1	9
**SARC**	ADPGK/ EI24/ RPS4Y1/ STRN/ TP53/ ZDHHC15	6
**SKCM**	CRYBA1/ CSAG1/ DDX3Y/ EIF1AY/ HOXB2/ KDM5D/ MAGEA12/ MAGEA3/ MAGEA6/ PPAPDC3/ RPS27/ SLC35E2/ UTY	14
**STAD**	OR2G6/ SLC35E2/ ZNF597	3

Tumor	Bimodal genes	Total
**TGCT**	ACACA/ ACTR8/ AGPAT6/ ALDOA/ ANXA5/ AP1M2/ ATF1/ BECN1/ C14orf179/ C5orf43/ C6orf192/ CCDC117/ CCT3/ CEP290/ CHN1/ CLPTM1/ CNOT8/ CTNNA1/ DCUN1D5/ DDX43/ DRAM2/ EHMT2/ EIF4B/ EXOC7/ FMN2/ FUBP3/ GATAD2A/ INF2/ KDSR/ KHDRBS1/ LOC100286793/ MAP2K4/ MAP4K1/ MID1/ MYBL2/ NAA11/ NAA25/ NCOA6/ NDUFV2/ NIPSNAP1/ NLE1/ NSMAF/ OSTC/ PAPSS2/ PCMTD1/ PDCD2/ PERP/ PFKM/ POLR3B/ POU5F1/ POU5F1B/ PQLC3/ PREX1/ PVRL2/ RAB34/ RAB5C/ RMND1/ RNF138/ RTKN/ SAP18/ SGK223/ SHC1/ SLC35E2/ SLC43A1/ SOX15/ STT3A/ SYCP3/ TCP10L2/ UGP2/ XPNPEP1/ ZCCHC8/ ZNF282/ ZNF304/ ZNF330/ ZNF526/ ZNF610/ ZNF766	77
**THCA**	ALDH3B1/ ANXA1/ BID/ C17orf54/ CYorf15A/ DDX3Y/ EIF1AY/ ERBB3/ FAM176A/ FAM71C/ FLJ42709/ GALE/ GBP2/ GJB3/ GRB7/ GSTM1/ GSTT1/ IL1RAP/ ITGB8/ KDM5D/ LGALS3/ LLGL1/ LQK1/ LRRC8A/ LY6E/ MDFIC/ MXRA8/ NGEF/ NLRP2/ NOD1/ PALM/ PDLIM4/ PLAG1/ PLCD3/ PRR25/ PTPRE/ PVRL4/ RASGEF1B/ RND3/ RPL9/ RPS27/ RPS28/ RPS4Y1/ RUNX2/ SCNN1A/ SDC4/ SLC35E2/ TMEM43/ TMSB4Y/ TNFRSF12A/ TTTY15/ TYW1B/ USP9Y/ UTY/ ZFY	55
**THYM**	ABT1/ ACTG1/ AEBP1/ AGAP2/ ANAPC16/ ARHGAP15/ ARHGAP33/ ARHGAP39/ ARHGDIB/ ASF1B/ ASRGL1/ AURKB/ BLM/ C14orf64/ C15orf44/ C16orf52/ C1orf172/ C21orf58/ C21orf96/ C2orf29/ C6orf115/ C8orf76/ CALM2/ CBFA2T3/ CCDC109B/ CCNB1/ CCNF/ CD3E/ CDC25C/ CDCA2/ CDCA3/ CDK6/ CDKN2C/ CDKN2D/ CENPA/ CENPH/ CENPL/ CENPN/ CENPW/ CERKL/ CHST2/ CKS2/ CNTROB/ COQ10A/ CSNK2A1P/ CSTF3/ DDX3Y/ DEPDC1/ DIAPH3/ DMC1/ DNAJC9/ DNTT/ E2F2/ EIF1AY/ ERCC6L/ EVL/ EWSR1/ EXO1/ FEN1/ FICD/ FOXRED1/ GAL3ST4/ GIT2/ GLE1/ GNA15/ GNG2/ GTF2I/ H1FX/ H2AFV/ HAUS1/ HDAC7/ HDGF/ HHIP/ HNRNPD/ HNRNPL/ HSD17B11/ IDH2/ IL10RB/ ILF2/ KDM5D/ KHDRBS1/ KIF2C/ KPNA2/ KPTN/ LAIR1/ LDHB/ LIG1/ LOC100128191/ LOC100189589/ LOC153684/ LOC257358/ LRRC8D/ LYAR/ MAGOH/ MAP1A/ MAZ/ MBD4/ MCM10/ MCM6/ MLLT11/ MND1/ MPP1/ MRPL55/ NAIP/ NASP/ NSMCE2/ NUCB2/ NUP88/ NUP93/ OIP5/ PBK/ PCNA/ PDCD2/ PHGDH/ PHPT1/ PLCD3/ PPIL5/ PPM1K/ PPP2R5D/ PRC1/ PRKY/ PSRC1/ PTP4A2/ PTPN7/ PVRIG/ R3HDM1/ RAC2/ RALY/ RBBP7/ RELL2/ RLTPR/ RNASEH2B/ RPF1/ RPIA/ RPS4Y1/ SAFB/ SBK1/ SEC31B/ SEH1L/ SF3B2/ SGTA/ SIT1/ SKA1/ SMARCB1/ SMPD3/ SNHG3-RCC1/ SOX4/ SPC25/ SPIN3/ SPN/ STMN1/ SURF6/ TCF19/ TCF7/ TDP1/ TMEM218/ TMEM39B/ TMEM70/ TMSB4Y/ TOE1/ TOR2A/ TRA2B/ TRAF3IP3/ TREML2/ TSNARE1/ TTC7A/ TUBA1B/ TUBB/ TUBGCP4/ UBE2I/ UBR7/ UBTF/ UCP2/ UHRF1/ USP8/ UTP3/ UTY/ VPS26B/ VRK1/ WDR62/ ZFY	181
**UCEC**	ALKBH3/ C2orf43/ EPM2AIP1/ EYA2/ HNF1B/ LDOC1/ MLH1/ RPS26P11/ SLC35E2/ SPAG16/ TUSC1	11
