# Supplementary material for: An integrated approach to identify bimodal genes associated with prognosis in câncer
Source: Genet Mol Biol. 2021 Oct 4;44(3):e20210109. doi: 10.1590/1678-4685-GMB-2021-0109 (PMC8495773; doi:10.1590/1678-4685-GMB-2021-0109)
Supplement: Figure S1 - [file 1415-4757-GMB-44-3-e20210109-s2.pdf]

Genetics and Molecular Biology
https://doi.org/10.1590/1678-4685-GMB-2021-0109

"Supplementary Material to "An integrated approach to identify bimodal genes associated with prognosis in cancer"

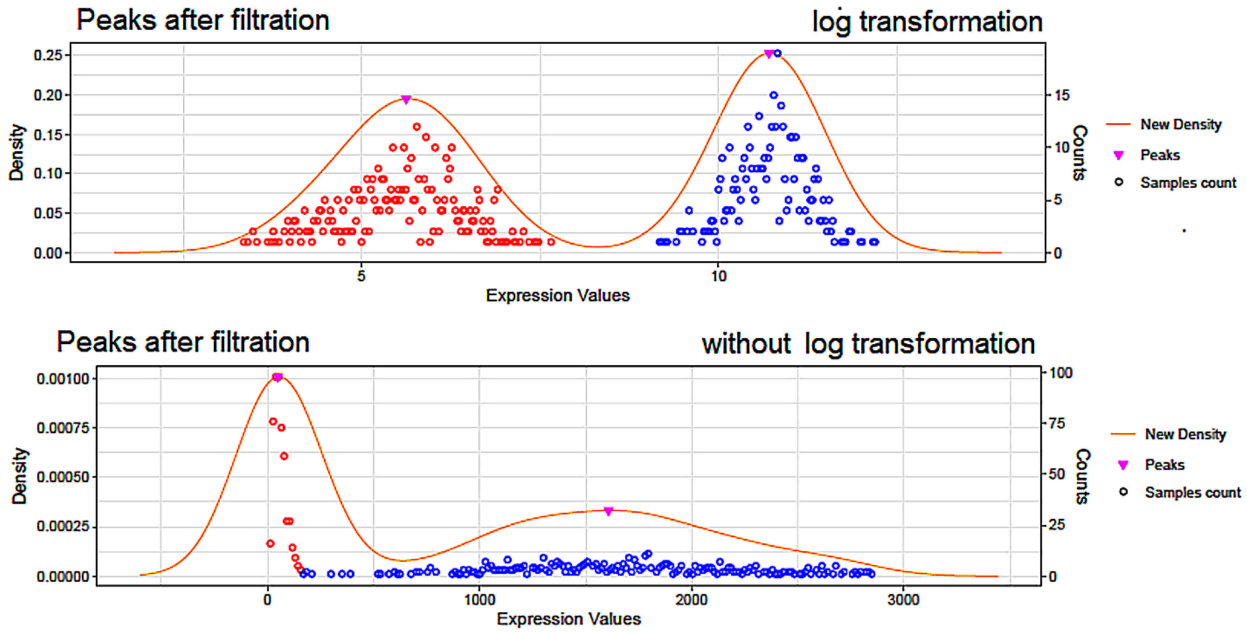

Figure S1 - Detection of bimodality for the RPS27 gene using gene expression data for breast adenocarcinoma (BRCA) from TCGA applying (upper graph), or not (lower graph), the log_{10} transformation to the data.
