# Supplementary material for: An integrated approach to identify bimodal genes associated with prognosis in câncer
Source: Genet Mol Biol. 2021 Oct 4;44(3):e20210109. doi: 10.1590/1678-4685-GMB-2021-0109 (PMC8495773; doi:10.1590/1678-4685-GMB-2021-0109)
Supplement: Figure S2 - [file 1415-4757-GMB-44-3-e20210109-s3.pdf]

Genetics and Molecular Biology
https://doi.org/10.1590/1678-4685-GMB-2021-0109

"Supplementary Material to "An integrated approach to identify bimodal genes associated with prognosis in cancer"

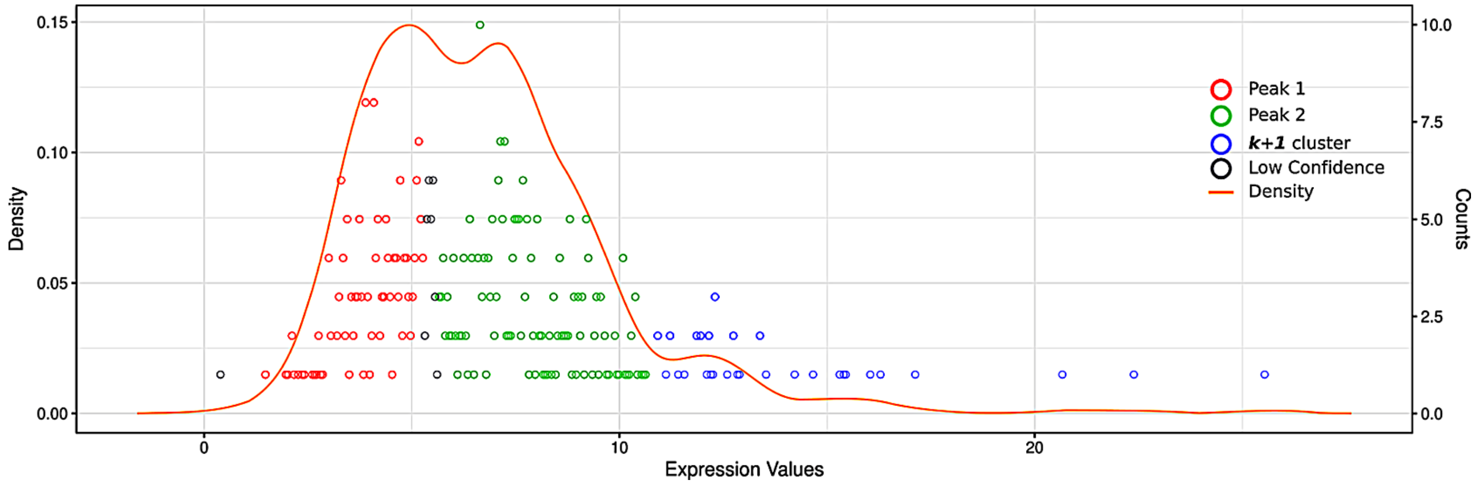

Figure S2 - Clustering of samples in k + 1 clusters. Red circles correspond to count of samples belonging to the first mode of expression. Green circles correspond to count of samples samples belonging to the second mode of expression. Blue circles correspond to outlier samples.
